# On the Extremal Wiener Polarity Index of Hückel Graphs

**DOI:** 10.1155/2016/3873597

**Published:** 2016-05-09

**Authors:** Hongzhuan Wang

**Affiliations:** Faculty of Mathematics and Physics, Huaiyin Institute of Technology, Huai'an, Jiangsu 223003, China

## Abstract

Graphs are used to model chemical compounds and drugs. In the graphs, each vertex represents an atom of molecule and edges between the corresponding vertices are used to represent covalent bounds between atoms. The* Wiener polarity index W*_*p*_(*G*) of a graph *G* is the number of unordered pairs of vertices *u*, *v* of *G* such that the distance between *u* and *v* is equal to 3. The trees and unicyclic graphs with perfect matching, of which all vertices have degrees not greater than three, are referred to as the* Hückel trees* and* unicyclic Hückel graphs*, respectively. In this paper, we first consider the smallest and the largest Wiener polarity index among all Hückel trees on 2*n* vertices and characterize the corresponding extremal graphs. Then we obtain an upper and lower bound for the Wiener polarity index of unicyclic Hückel graphs on 2*n* vertices.

## 1. Introduction

Nearly half a century ago, the development of quantum chemistry is largely due to the wide application of the concept of graph. One of the major topics in this field is molecular topological index. The molecular topological index can describe the structure of the molecule quantitatively, as an invariant of the graph can be used to demonstrate the relationship between the molecules structure and performance. Quantitative structure activity relationships are a popular computational biology paradigm in modern drug design.

One of the most widely known topological descriptors is Wiener polarity index. The Wiener polarity index of an organic molecule graph of which *G* = (*V*(*G*), *E*(*G*)) is defined by

(1) $$\begin{array}{c} W_{p}\left(\;G\;\right)=\left|\;\left\{\;\left(\;u,v\;\right)\;|\;d_{G}\left(\;u,v\;\right)=3,\;\;u,v\in V\left(\;G\;\right)\;\right\}\;\right|, \end{array}$$

which is the number of unordered pairs of vertices *u*, *v* of *G* such that *d*_*G*_(*u*, *v*) = 3, where *d*_*G*_(*u*, *v*) denotes the distance between two vertices *u* and *v* in *G*.

The Wiener polarity index for the quantity defined in the equation above is introduced by Wiener [1] for acyclic molecules in a slightly different yet equivalent manner. Moreover, Wiener [1] used a linear formula for the Wiener index *W*≔∑_*u*,*v*⊆*V*_ *d*_*G*_(*u*, *v*) and the Wiener polarity index *W*_*p*_ to calculate the boiling points *t*_*B*_ of the paraffins; that is,

(2) $$\begin{array}{c} t_{B}=aW+bW_{p}+c, \end{array}$$

where *a*, *b*, and *c* are constants for a given isomeric graph.

In 1998, by using the Wiener polarity index, Lukovits and Linert [2] demonstrated quantitative structure-property relationships in a series of acyclic and cycle-containing hydrocarbons. Besides, a physical-chemical interpretation of *W*_*p*_(*G*) was found by Hosoya [3]. Recently, Du et al. [4] obtained the smallest and largest Wiener polarity indices together with the corresponding graphs among all trees on *n* vertices, respectively. Deng et al. [5] characterized the extremal Wiener polarity index of trees with a given diameter. The authors in [6] found the maximum Wiener polarity index among all chemical trees with *n* vertices and *k* pendents. Hou et al. [7] found the maximum Wiener polarity index of unicyclic graphs together with the corresponding extremal graphs.

As is well known, conjugated hydrocarbon molecules considered in the Hückel molecule orbit theory are usually represented by the carbon-atom skeleton graphs, of which all vertices have degrees less than four. We call such molecular graphs Hückel molecular graphs. In graph theory, the Hückel molecular graphs with Kekulé structures are graphs with perfect matchings of which the largest degree of vertices does not exceed three.

Let *N*_*G*_(*u*) be the neighbor vertex set of *u* in *G*. Then *d*_*G*_(*u*) = |*N*_*G*_(*u*)| is called the degree of *u*. If *d*_*G*_(*v*) = 1, then we call *v* a pendent vertex of *G*. Let Δ(*G*) denote the maximum vertices degree in *G*. As usual, let *C*_*n*_ and *P*_*n*_ be the cycle and path of order *n*, respectively. A path *P* in *G* is called *i*-*degree pendent chain* if all its internal vertices are of degree 2 and its ends of degrees 1 and *i*, respectively, where *i* ≥ 3. A* matching M* of the graph *G* is a subset of *E*(*G*) such that no two edges in *M* share a common vertex. If *M* is a matching of a graph *G* and vertex *v* is incident with an edge of *M*, then *v* is said to be *M*-*saturated*, and if every vertex of *G* is *M*-saturated, then *M* is a* perfect matching*. Suppose *uv* ∈ *E*(*G*); the notion *G* − *uv* denotes the new graph yielded from *G* by deleting the edge *uv*. Similarly, if *uv* ∉ *E*(*G*), then *G* + *uv* denotes the new graph obtained from *G* by adding the edge *uv*. The set of the Hückel trees and Hückel unicyclic graphs with 2*n* vertices is denoted by *𝒯*_2*n*_ and *ℋ*_2*n*_, respectively.

In the paper, we consider the Wiener polarity index for Hückel trees and Hückel unicyclic graphs. In Section 2, we discuss some properties of the Wiener polarity index of Hückel trees. In Section 3, we determine the smallest and largest Wiener polarity index together with the corresponding graphs among all Hückel trees. In Section 4, the smallest and the largest Wiener polarity indices among all Hückel unicyclic graphs on 2*n* vertices are identified, respectively.

## 2. Some Properties of the Wiener Polarity Index of Hückel Trees

In this section, first, we give some formulas for computing the Wiener polarity index of trees.

**Lemma 1 (see [4]).**

Let *T* = (*V*, *E*) be a tree. Then

(3) $$\begin{array}{c} W_{p}\left(\;T\;\right)=\underset{uv\in E\left(T\right)}{\sum }\left(\;d_{T}\left(\;u\;\right)-1\;\right)\left(\;d_{T}\left(\;v\;\right)-1\;\right). \end{array}$$

**Lemma 2 (see [8]).**

Let *T* be a 2*n*-vertex tree (*n* ≥ 2) with a perfect matching. Then *T* has at least two pendent vertices such that each is adjacent to vertices of degree two.

For any *T* ∈ *𝒯*_2*n*_, the following several lemmas will give necessary conditions on which *W*_*p*_(*T*) attains the maximum values.

**Lemma 3 .**

Let *T* be a graph in *𝒯*_2*n*_ such that *W*_*p*_(*T*) is as larger as possible. Then the lengths of all pendent chains in *T* are no more than 2.

**Proof**

By contradiction. Assume that there exists a pendent chain *P* = *u*_0_ *u*_1_ *u*_2_ ⋯ *u*_*k*−1_ *u*_*k*_ with length *k* such that *k* ≥ 3; we distinguish the following two cases.

*Case 1 (k* = 3). This implies that there exists a pendent chain *P* = *u*_0_ *u*_1_ *u*_2_ *u*_3_ such that *d*_*T*_(*u*_0_) = 3, *d*_*T*_(*u*_1_) = *d*_*T*_(*u*_2_) = 2, and *d*_*T*_(*u*_3_) = 1. We claim that the vertex adjacent to *u*_0_ cannot be a pendent vertex; suppose, on the contrary, that *v*_0_ is a pendent vertex adjacent to *u*_0_. Assuming that *M* is the perfect matching of *T*, we know that *M* is unique in trees and each pendent edge of *T* belongs to *M*; therefore, *u*_0_ *v*_0_ ∈ *M* and *u*_2_ *u*_3_ ∈ *M*. Then *u*_1_ is not saturated by *M*, a contradiction. Let *v* be a vertex of degree 2 nearest to *u*_0_ except for *u*_1_. Let *T*′ = *T* − *u*_1_ *u*_2_ + *vu*_2_, then obviously, *T*′ ∈ *𝒯*_2*n*_. The following two subcases should be considered.

*Subcase 1.1 (u*_0_ *v* ∈ *E*(*T*)). In this case, we assume that *a* is another neighbor of *v*; by Lemma 1, we have

(4) WpT′−WpT2dTa−1+4+2−2+2+1+dTa−1=dTa≥1>0.

It contradicts the maximality of *W*_*p*_(*T*).

*Subcase 1.2 (u*_0_ *v* ∉ *E*(*G*)). In this case, Let *a* and *b* be the neighbors of *v*; by Lemma 1, we have

(5) WpT′−WpT2dTa+dTb−2+2−dTa+dTb+1=dTa+dTb−3.

If *d*_*T*_(*a*) = 1, then *d*_*T*_(*b*) ≥ 2; otherwise, if *d*_*T*_(*b*) = 1, there are two pendent edges which are adjacent to vertex *v*, a contradiction to the fact there exists perfect matching. Furthermore, by the choice of *v*, we deduce that *d*_*T*_(*b*) ≠ 2; if not, *v* is not the vertex of degree 2 nearest to *u*_0_. If *d*_*T*_(*b*) = 3, we also have *W*_*p*_(*T*′) > *W*_*p*_(*T*), a contradiction once again.

*Case 2 (k* ≥ 4). Let *P* = *u*_0_ *u*_1_ *u*_2_ ⋯ *u*_*k*−1_ *u*_*k*_ be the pendent path with length *k*. Let *T*′ = *T* − *u*_*k*−2_ *u*_*k*−1_ + *u*_*k*−3_ *u*_*k*−1_, and then *T*′ ∈ *𝒯*_2*n*_; by Lemma 1, we have

(6) $$\begin{array}{c} W_{p}\left(\;T^{\prime }\;\right)-W_{p}\left(\;T\;\right)=\left(\;2+2\;\right)-\left(\;1+1+1\;\right)=1>0. \end{array}$$

Thus, *W*_*p*_(*T*′) > *W*_*p*_(*T*), a contradiction. This completes the proof.

By Lemma 3, we can show that if *T* ∈ *𝒯*_2*n*_ with maximum *W*_*p*_(*T*), the length of any pendent chain is either 2 or 1. Therefore, we have reduced the problem to the Hückel trees having a path with both ends of degree 3. Then, we introduce a graph transformation which will be used in the following proof.

Let *T* be a tree in *𝒯*_2*n*_ with *n* ≥ 2. Let *e* = *uv* be a nonpendent edge of *T*. *T*_1_ and *T*_2_ are two components of *T* − *e*, *u* ∈ *T*_1_, and *v* ∈ *T*_2_. *T*_0_ is the graph obtained from *T* in the following way:

(1) Contract the edge *e* = *uv* (i.e., identify *u* of *T*_1_ with *v* of *T*_2_).
(2) Add a pendent edge to the vertex *u*( = *v*).

**Figure 1:**
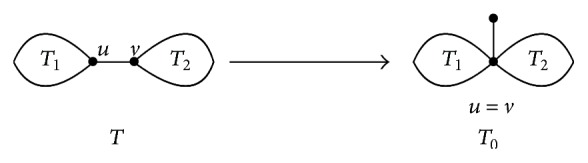
The edge-growth transformation.

We call procedures (1) and (2) the edge-growth transformation of *T* or e.g.t of *T* for short (see Figure 1).

**Lemma 4 .**

Let *T* be a graph in *𝒯*_2*n*_ such that *W*_*p*_(*T*) is as larger as possible. If *P* is a path in *T* with two end-vertices of degree 3, then all internal vertices of *P* are of degree 3.

**Proof**

Suppose, on the contrary, that there is a path *xv*_1_ ⋯ *v*_*t*_ *y* in *T* such that *d*_*T*_(*x*) = 3, *d*_*T*_(*y*) = 3, *d*_*T*_(*v*_1_) = ⋯ = *d*_*T*_(*v*_*t*_) = 2, and *t* ≥ 1. Let *M* be the perfect matching of *T*; we consider the following two cases.

*Case 1 (t is even)*. In this case, it is easy to see that either *xv*_1_, *v*_*t*_ *y* ∉ *M* or *xv*_1_, *v*_*t*_ *y* ∈ *M*. If not, there must exist a vertex of path *v*_1_ ⋯ *v*_*t*_ not saturated by *M*. We distinguish the following subcases.

*Subcase 1.1 (xv*_1_ ∉ *M and v*_*t*_ *y* ∉ *M*). Since *t* is even, the vertices *v*_1_, *v*_2_,…, *v*_*t*_ of path *xv*_1_ ⋯ *v*_*t*_ *y* are matched mutually. That is to say, *v*_1_ *v*_2_ ∈ *M*, *v*_3_ *v*_4_ ∈ *M*,…, *v*_*t*−1_ *v*_*t*_ ∈ *M*. One can transform *T* into *T*′ by using exactly *t*/2 steps of e.g.t for above edges continuously; we note that the resulting graph *T*′ is a tree obtained by attaching one pendent edge to each vertex of *v*_1_, *v*_3_,…, *v*_*t*−1_. Then *T*′ ∈ *𝒯*_2*n*_. Then by Lemma 1, we have

(7) WpT′−WpT4t−22+2−t−1+2+2=t+1>0,

which contradicts the maximality of *W*_*p*_(*T*).

**Figure 2:**
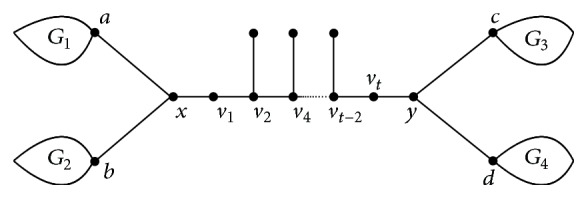
The trees in class (I).

*Subcase 1.2 (xv*_1_ ∈ *M and v*_*t*_ *y* ∈ *M*). In this subcase, we can easily see that the vertices *v*_2_, *v*_3_, *v*_4_,…, *v*_*t*−2_, *v*_*t*−1_ of *P* are mutually matched. That is to say, *v*_2_ *v*_3_ ∈ *M*, *v*_4_ *v*_5_ ∈ *M*,…, *v*_*t*−2_ *v*_*t*−1_ ∈ *M*; then one can transform *T* into *T*′ by using exactly (*t* − 2)/2 steps of e.g.t continuously. We notice that the resulting graph *T*′ is a Hückel tree obtained by attaching one pendent edge to each vertex of *v*_2_, *v*_4_,…, *v*_*t*−2_. Then *T*′ is one of class (I) of trees, as shown in Figure 2.

Let *a* and *b* be two neighbors of vertex *x* and *c* and *d* be two neighbors of *y*, respectively. Let *G*_1_, *G*_2_, *G*_3_, and *G*_4_ be the connected components containing *a*, *b*, *c*, and *d* of the graph *T* − *x* − *y*, respectively. Also, by Lemma 1, we have

(8) WpT′−WpTt−42×4+2+2−t−1=t−3.

If *t* > 3, *W*_*p*_(*T*′) > *W*_*p*_(*T*). If *t* = 2, *T* is also one of class (I) of trees.

In the following, we have reduced the problem to the Hückel trees of class (I). For *xv*_1_ ∈ *M*, then *ax* and *bx* cannot be pendent edges of *T*′, since *T*′ ∈ *𝒯*_2*n*_. There are at least two vertices in *G*_1_ and *G*_2_; without loss of generality, we consider *G*_1_. Let *n*_*i*_(*G*_*i*_) be the number of vertices of *G*_*i*_. We distinguish the following subcases.

*Subcase 1.2.1 (n*_1_(*G*_1_) = 2). Let *wa* be pendent edge of *G*_1_, where *w* is pendent vertex. Let *T*′′ = *T*′ − *aw* + *v*_1_ *w*. Denote *M*′ to be the perfect matching of *T*′; then *M*′′ = *M*′ − *xv*_1_ − *wa* + *ax* + *v*_1_ *w* is the perfect matching of *T*′′; we notice that *T*′′ ∈ *𝒯*_2*n*_, and by Lemma 1, there is

(9) $$\begin{array}{c} W_{p}\left(\;T^{\prime \prime }\;\right)-W_{p}\left(\;T^{\prime }\;\right)=\left(\;4+4\;\right)-\left(\;2+2+2\;\right)=2>0. \end{array}$$

Therefore, *W*_*p*_(*T*′′) > *W*_*p*_(*T*′) > *W*_*p*_(*T*), a contradiction.

*Subcase 1.2.2 (n*_1_(*G*_1_) ≥ 3). Obviously, *G*_1_ is a subgraph of *T*′ with a perfect matching, since *xv*_1_ ∈ *M*. Then by Lemma 2, there exists a pendent vertex *v* which is adjacent to *u* of degree 2; let *w* be another neighbor of *u* in *T*′. Let *T*′′ = *T*′ − *wu* + *v*_1_ *u*; it is easy to see that *M*′ is still the perfect matching of *T*′′, and then *T*′′ ∈ *𝒯*_2*n*_; by Lemma 1, we have

(10) WpT′′−WpT=10+∑xi∈NT′w∖udT′w−2dT′xi−1−5+∑xi∈NT′w∖udT′w−1dT′xi−1=5−∑xi∈NT′w∖udT′xi−1.

From Lemma 3, it is noted that *d*_*T*′_(*w*) = 3; otherwise, if *d*_*T*′_(*w*) = 2, then there exists a pendent chain with length of at least 3, a contradiction. Let *x*_1_ and *x*_2_ denote the two neighbors of *w* in *G*_1_; it should be noted that 1 ≤ *d*_*T*′_(*x*_1_) ≤ 3 and 1 ≤ *d*_*T*′_(*x*_2_) ≤ 3. Then by Lemma 1, we have

(11) WpT′′−WpT′5−∑xi∈NT′w∖udT′xi−1≥7−dT′x1−dT′x2≥1.

Therefore, *W*_*p*_(*T*′′) > *W*_*p*_(*T*′) > *W*_*p*_(*T*), a contradiction.

The analysis on *v*_*t*_ of degree 2 is the same as that for *v*_1_.

**Figure 3:**
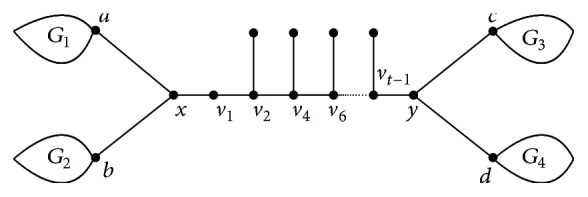
The trees in class (II).

*Case 2 (t is odd)*. In this case, there are odd vertices of degree 2 in the path *xv*_1_ ⋯ *v*_*t*_ *y*; then there exits exactly one of two edges *xv*_1_ and *v*_*t*_ *y* which belongs to *M*; without loss of generality, we assume that *xv*_1_ ∈ *M*. It should be noted that *v*_2_ *v*_3_ ∈ *M* and *v*_4_ *v*_5_ ∈ *M*,…, *v*_*t*−1_ *v*_*t*_ ∈ *M*; then one can transform *T* into *T*′ by using exactly (*t* − 1)/2 steps of e.g.t continuously. We notice that the resulting graph *T*′ is a Hückel tree obtained by attaching one pendent edge to each vertex of *v*_2_, *v*_4_,…, *v*_*t*−1_; then *T*′ is one of class (II), as shown in Figure 3.

We also notice that

(12) WpT′−WpT4×t−32+2+4−t−2+1+2=t−1.

If *t* > 2, there is *W*_*p*_(*T*′) > *W*_*p*_(*T*). If *t* = 1, *T* is also in class (II) of trees. The proof is similar to that of Subcase 1.2.

In any case, the resulting graph belongs to *𝒯*_2*n*_ such that all the internal vertices of the path *xv*_1_ ⋯ *v*_*t*_ *y* are of degree 3. Furthermore, the resulting graph has the value of Wiener polarity no less than that of *T*, which contradicts the maximality of *W*_*p*_(*T*).

This completes the proof.

The next result follows obviously from the proof of Lemmas 3 and 4.

**Corollary 5 .**

Let *T* ^*∗*^ have maximal Wiener polarity index in *𝒯*_2*n*_ (*n* ≥ 3). Then there exist the following properties of *T* ^*∗*^:

(i) All the lengths of pendent chains are no more than 2.
(ii) If *P* is a path in *T* ^*∗*^ with both ends of degree 3, then all internal vertices of *P* are of degree 3.
(iii) All the vertices of degree 2 in *T* ^*∗*^ are on the pendent chains.

## 3. The Extremal Wiener Polarity Index of Hückel Trees

In this section, we will discuss the maximum and minimum Wiener polarity index of Hückel trees with 2*n* vertices. Firstly, we consider the Hückel trees with the largest Wiener polarity index.

Let *m*_*ij*_ be the number of edges in *T* between vertices of degrees *i* and *j*. By Lemma 1, we have

(13) WpT∑uv∈ETdTu−1dTv−1=∑1≤i≤j≤n−1i−1j−1mij.

In particular, if *T* is a Hückel tree, then

(14) $$\begin{array}{c} W_{p}\left(\;T\;\right)=m_{22}+2m_{23}+4m_{33}. \end{array}$$

Let *T* ^*∗*^ ∈ *𝒯*_2*n*_ with a vertices sequence (*n*_1_, *n*_2_, *n*_3_), where *n*_*i*_ denotes the number of vertices of *T* ^*∗*^ with degree *i*. Recall the following relations:

(15) n1+n2+n3=2n,n1+2n2+3n3=4n−2.

From above two equalities it follows that

(16) n1=n3+2,n2=2n−2n3−2.

By Corollary 5, it should be noted that the subgraph *T*′ induced by the vertices of degree 3 in *T* ^*∗*^ is also a tree. Then we deduce that

(17) m22=0,m23=n2,m33=n3−1.

Then, by Corollary 5, we have

(18) $$\begin{array}{c} W_{p}\left(\;T^{*}\;\right)=m_{22}+2m_{23}+4m_{33}=2n_{2}+4\left(\;n_{3}-1\;\right). \end{array}$$

By above equations, we have that *W*_*p*_(*T* ^*∗*^) = 2(2*n* − 2*n*_3_ − 2) + 4(*n*_3_ − 1) = 4*n* − 8. From Corollary 5 and the arguments above, the following result is obvious.

**Theorem 6 .**

Suppose *T* is a graph in *𝒯*_2*n*_ with *n* ≥ 3. Then *W*_*p*_(*T*) ≤ 4*n* − 8, and equality holds if and only if *T*≅*T* ^*∗*^.

Next, we consider the minimum Wiener polarity index among *𝒯*_2*n*_, and we first consider some special cases.

If *n* = 1, *T*_2*n*_≅*P*_2_ and *W*_*p*_(*P*_2_) = 0; If *n* = 2, *T*_2*n*_≅*P*_4_ and *W*_*p*_(*P*_4_) = 1.

In the following, we assume that *n* ≥ 3.

For all Hückel trees *T* in *𝒯*_2*n*_, sharp lower bounds for *W*_*p*_(*T*) are obtained in the following theorem.

**Theorem 7 .**

Suppose *T* is a graph in *𝒯*_2*n*_ with *n* ≥ 3, then *W*_*p*_(*T*) ≥ 2*n* − 3, and equality holds if and only if *T*≅*P*_2*n*_.

**Proof**

**Figure 4:**
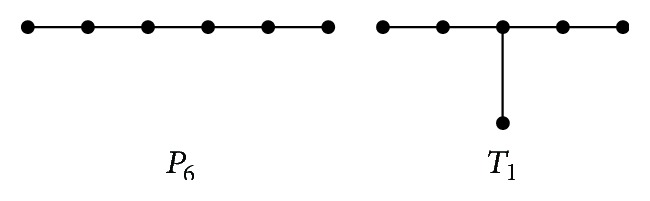
Hückel trees with 2*n* = 6.

We prove the assertion by induction on *n*. If *n* = 3, then *T*≅*P*_6_ or *T*≅*T*_1_ (see Figure 4). It can be easily checked that *W*_*p*_(*P*_6_) = 3 < *W*_*p*_(*T*_1_) = 4. The result holds for *n* = 3.

Now assume the assertion holds for all Hückel trees with less than *n* ≥ 4 vertices. Suppose *T* is a Hückel tree with 2*n* vertices; by Lemma 2, then there exists a pendent vertex *v* which is adjacent to *u* of degree 2. Let *T*′ = *T* − *u* − *v*; it should be noted that *T*′ ∈ *𝒯*_2*n*−2_. Let *u*′ be adjacent to *u* of *T*; that is, *uu*′ ∈ *E*(*T*). We distinguish the following cases.

*Case 1 (d*_*T*_(*u*′) = 2). Let *a* be another neighbor of *u*′; then by Lemma 1, we have *W*_*p*_(*T*) − *W*_*p*_(*T*′) = (*d*_*T*_(*a*) − 1) + 1 = *d*_*T*_(*a*) ≥ 2, since 2*n* ≥ 6, by induction hypothesis, so *W*_*p*_(*T*) ≥ *W*_*p*_(*T*′) + 2 ≥ 2(*n* − 1) − 3 + 2 = 2*n* − 3, with equality if and only if *T*′≅*P*_2*n*−2_ and *d*_*T*_(*a*) = 2. Now we reconstruct the tree *T* from *T*′≅*P*_2*n*−2_ by attaching a pendent chain with length 2 to the vertex *u*′. It follows that *T* ∈ *𝒯*_2*n*_. Hence, *W*_*p*_(*T*) ≥ 2*n* − 3, and the equality holds if and only if *T*≅*P*_2*n*_.

*Case 2 (d*_*T*_(*u*′) = 3). Let *u*_1_′ and *u*_2_′ be the other two neighbors of *u*′; then by Lemma 1, we have

(19) WpT−WpT′dTu1′−1+dTu2′−1+2=dTu1′+dTu2′.

That is to say, *W*_*p*_(*T*) = *W*_*p*_(*T*′) + *d*_*T*_(*u*_1_′) + *d*_*T*_(*u*_2_′). If *d*_*T*_(*u*_1_′) = 1, then *d*_*T*_(*u*_2_′) ≥ 2; if not, there is no perfect matching in *𝒯*_2*n*_, and by induction hypothesis, we have

(20) $$\begin{array}{c} W_{p}\left(\;T\;\right)\geq 2\left(\;n-1\;\right)-3+1+2=2n-3+1>2n-3. \end{array}$$

The result holds.

## 4. The Wiener Polarity Index of Unicyclic Hückel Graphs

In this section, we will give sharp lower and upper bounds for Wiener polarity index of unicyclic Hückel graphs. The girth *g*(*G*) of a connected graph *G* is the length of shortest cycle in *G*.

First, we will establish some lemmas which will be useful to the proofs of our main results.

**Lemma 8 (see [9]).**

Let *U* = (*V*, *E*) be a unicyclic graph. If *g*(*U*) = 3 with *V*(*C*_3_) = {*v*_1_, *v*_2_, *v*_3_}, then

(21) WpU=∑uv∈EUdUu−1dUv−1+9−2dUv1−2dUv2−2dUv3;

if *g*(*U*) = 4 with *V*(*C*_4_) = {*v*_1_, *v*_2_, *v*_3_, *v*_4_}, then

(22) WpU=∑uv∈EUdUu−1dUv−1+4−dUv1−dUv2−dUv3−dUv4.

 Moreover, if *g*(*U*) = 5, *W*_*p*_(*U*) = ∑_*uv*∈*E*(*U*)_(*d*_*U*_(*u*) − 1)(*d*_*U*_(*v*) − 1) − 5;
 if *g*(*U*) = 6, *W*_*p*_(*U*) = ∑_*uv*∈*E*(*U*)_(*d*_*U*_(*u*) − 1)(*d*_*U*_(*v*) − 1) − 3;
 if *g*(*U*) ≥ 7, *W*_*p*_(*U*) = ∑_*uv*∈*E*(*U*)_(*d*_*U*_(*u*) − 1)(*d*_*U*_(*v*) − 1).

**Lemma 9 .**

Let *U* be a unicyclic Hückel graph with 2*n* vertices. Then

(1) if *g*(*U*) = 3, then 2*n* − 4 ≤ *W*_*p*_(*U*) ≤ 4*n* − 5;
(2) if *g*(*U*) = 4, then 2*n* − 4 ≤ *W*_*p*_(*U*) ≤ 4*n* − 4.

**Proof**

We only prove the first assertion, and the second assertion can be proved analogously. Let *U* be a unicyclic Hückel graph with *g*(*U*) = 3 and *v*, *w*, and *u* be the three vertices on the unique cycle of *U*, and let *d*_*U*_(*v*) = *k*_1_ + 2, *d*_*U*_(*u*) = *k*_2_ + 2, and *d*_*U*_(*w*) = *k*_3_ + 2. *N*_*U*_(*v*) = {*w*, *u*, *v*_1_, *v*_2_,…, *v*_*k*_1__}, *N*_*U*_(*u*) = {*w*, *v*, *u*_1_, *u*_2_,…, *u*_*k*_2__}, and *N*_*U*_(*w*) = {*v*, *u*, *w*_1_, *w*_2_,…, *w*_*k*_3__}, where 0 ≤ *k*_1_, *k*_2_, *k*_3_ ≤ 1.

Let *M* be the perfect matching of *U*; there exists one edge on the unique cycle of *U* that does not belong to *M*; otherwise, there is a contradiction to the fact that *U* has perfect matching; without loss of generality, suppose that *wu* ∉ *M*, and then deleting the edge *wu*, we get a Hückel tree *T* and *M* still is the perfect matching of *T*. By Lemmas 1 and 8, we have

(23) WpU−WpT=∑uv∈EUdUu−1dUv−1+9−2dUu−2dUv−2dUw−∑uv∈ETdTu−1dTv−1=dUu1−1+dUu2−1+⋯+dUuk2−1+dUw1−1+⋯+dUwk3−1+k1+1k3+1−k3+k1+1k2+1−k2+k2+1k3+1+9−2k1+2−2k2+2−2k3+2=dUu1+dUu2+⋯+dUuk2+dUw1+⋯+dUwk3+k2k3−2k2−2k3.

Since *U* is a unicyclic Hückel graph, without loss of generality, we may assume that 0 ≤ *k*_2_ ≤ *k*_3_ ≤ 1. Then

(24) WpU−WpT=0;k2=k3=0,WpU−WpT=dUw1−2≤1;k2=0,  k3=1,WpU−WpT≤3,k2=k3=1.

Hence, by Theorem 6, we obtain that

(25) $$\begin{array}{c} W_{p}\left(\;U\;\right)\leq W_{p}\left(\;T\;\right)+3\leq 4n-5. \end{array}$$

Similarly, by Theorem 7, we obtain that

(26) $$\begin{array}{c} W_{p}\left(\;U\;\right)\geq W_{p}\left(\;T\;\right)-1\geq 2n-3-1=2n-4. \end{array}$$

This completes the proof.

**Lemma 10 .**

Let *U* be a unicyclic Hückel graph with 2*n* vertices. Then

(1) if *g*(*U*) ≥ 7, then 2*n* − 2 ≤ *W*_*p*_(*U*) ≤ 4*n* + 4;
(2) if *g*(*U*) = 6, then 2*n* − 5 ≤ *W*_*p*_(*U*) ≤ 4*n* + 1;
(3) if *g*(*U*) = 5, then 2*n* − 7 ≤ *W*_*p*_(*U*) ≤ 4*n* − 1.

**Proof**

We only prove the first assertion and other assertions can be proved similarly. Let *U* be a unicyclic Hückel graph with *g*(*U*) ≥ 7. Let *M* be the perfect matching of *U*; then there exists edge *uv* ∈ *E*(*C*_*k*_) such that *uv* ∉ *M*. We can get a Hückel tree *T* by deleting *uv*. By Lemma 8, we have

(27) WpU=WpT+kl+∑i=1kdUui+∑j=1ldUvj−k−l,

where *d*_*U*_(*u*) = *k* + 1, *d*_*U*_(*v*) = *l* + 1, *N*_*U*_(*u*) = {*v*, *u*_1_,…, *u*_*k*_}, and *N*_*U*_(*v*) = {*u*, *v*_1_,…, *v*_*l*_}; without loss of generality, assume that 1 ≤ *k* ≤ *l* ≤ 2; then

(28) WpU−WpT=dUu1+dUv1−1≤5;k=l=1,WpU−WpT=dUu1+∑i=12dUvi−1≤8;k=1,  l=2,WpU−WpT=∑i=12dUui+∑i=12dUvi≤12,k=l=2.

This completes the proof.

Combining Lemmas 9 and 10, we have the following result.

**Theorem 11 .**

Let *U* be a unicyclic Hückel graph in *ℋ*_2*n*_ with *n* ≥ 4. Then

(29) $$\begin{array}{c} 2n-7\leq W_{p}\left(\;U\;\right)\leq 4n+4. \end{array}$$

## 5. Conclusion

This paper determined the smallest and the largest Wiener polarity index among all Hückel trees and unicyclic Hückel graphs on 2*n* vertices and characterized the corresponding extremal graphs. Thus, the promising prospects of the application for the chemical and pharmacy engineering will be illustrated in the theoretical conclusion that is obtained in this paper.
